# Fluorinated Montmorillonite Composite Resin as a Dental Pit and Fissure Sealant

**DOI:** 10.3390/polym11101535

**Published:** 2019-09-20

**Authors:** Keng-Yuan Li, Cheng-Chia Tsai, Chih-Hsiang Fang, Yin-Lin Wang, Feng-Huei Lin, Chun-Pin Lin

**Affiliations:** 1 Institute of Biomedical Engineering, National Taiwan University, Taipei 106, Taiwan; d03548006@ntu.edu.tw (K.-Y.L.); danny07291991@gmail.com (C.-H.F.); 2 Department of Neurosurgery, Mackay Memorial Hospital, Taipei 104, Taiwan; angle@mmh.org.tw; 3 Graduate Institute of Clinical Dentistry, School of Dentistry, National Taiwan University, Taipei 100, Taiwan; 4 National Taiwan University Hospital, College of Medicine, National Taiwan University, Taipei 100, Taiwan; 5 Institute of Biomedical Engineering and Nanomedicine, National Health Research Institutes, Miaoli 350, Taiwan

**Keywords:** fluoride, composite resin, pit, fissure, sealant, bis-GMA, montmorillonite, dental caries

## Abstract

Molar pits and fissures tend to be affected by caries due to cleaning difficulties. As such, the filling of pits and cracks with sealants is common to deter the onset of caries. However, current clinical practices rely on sealants that lack the ability to release and recharge fluoride ions. Thus, we herein report the development of a fluoride—montmorillonite nanocomposite resin that has the potential to provide sustained release of fluoride due to the strong adsorption of fluoride by montmorillonite. X-ray diffractometry, thermogravimetric analysis, and Fourier-transform infrared spectroscopy were employed to confirm the successful insertion of the polymer into the interlayer structure. The mechanical properties (viscosity, hardening depth, hardness, diametral tensile strength, flexural strength, and wear resistance) of the developed composite resin were then examined, and simulation of the oral environment demonstrated a good fluoride ion release and recharge ability for the effective prevention of dental caries. Finally, we demonstrated the non-cytotoxic nature of this material using the water-soluble tetrazolium salt (WST-1) test. We expect that the described fluoride-containing composite resin may become a new clinical option in the near future.

## 1. Introduction

The fissures of permanent molars tend to be the first tooth surfaces to become affected by caries in younger age groups [1], with approximately 90% of carious lesions being found in the pits and fissures of permanent posterior teeth [2]. The cracks are typically narrow and deep, making them difficult to clean, and resulting in the formation of caries due to the accumulation of microorganisms and bacteria [3]. At present, the most commonly-employed clinical treatment for such caries is etching of the teeth with acid, followed by filling with a sealant to create a physical barrier against food and bacteria [4]. This method was first described by Buonocore in 1955 [5,6], and has since led to the development of a range of sealants [7,8].

Fluoride is considered the most effective treatment for fighting dental carries, as fluoride ions have been shown to reduce acid production [9,10]. As the potential parameter of fluoride is higher than that of OH^−^, fluoride can replace the OH groups on hydroxyapatite (HAP) to give fluorapatite (FAP), thereby reinforcing and strengthening the teeth [11,12]. As such, the fluoride ions present in oral treatments can both reduce demineralization and promote the remineralization of teeth [13]. Recent studies have indicated that if restoration materials contain fluoride, not only can demineralization and caries development be inhibited, but the neighboring tooth enamel and dentin can also be strengthened [14], thereby reducing the chance of tooth decay.

Currently, a number of sealant products are available that exhibit fluoride ion release; however, no sealer material has been developed that can balance the retention rate with fluoride release [15]. According to previous studies, the use of a high-viscosity glass-ionomer cement as a pit and fissure sealant increases the interproximal fluoride concentration to a greater extent than resin-based fluoride-containing sealants [16,17]. In addition, no sealant exists that is based on a resin matrix exhibiting both fluoride ion release and recharge. We; therefore, wish to address these issues in our study by developing a novel sealant that exhibits fluoride release and recharge properties.

Bis-GMA (bisphenol A-glycidyl methacrylate) can be synthesized from the diglycidyl ether of bisphenol A (DGEBA) and methacrylic acid, and was first prepared in 1962 by Bowen [18,19]. Compared to other materials, the polymerization shrinkage of Bis-GMA is less pronounced, it exhibits a low tissue diffusivity, a lower volatility, and it is easy to manipulate. In addition, its flow and mechanical properties can be easily controlled by altering the diluent: filler ratio [20], and so commercial composite resin products are commonly based on the bis-GMA system. However, the higher viscosity of bis-GMA renders the mixing of filler and monomer more challenging, including mixing of the chemically-cured composite. To address this problem, different monomers with lower viscosities (e.g., triethylene glycol dimethacrylate (TEGDMA) [21,22]) have been employed to dilute the highly-viscous bis-GMA monomer and allow the incorporation of greater quantities of filler.

Montmorillonite (MMT) is a layered aluminosilicate of the 2:1 type that is formed by two tetrahedral sheets of silicon oxide fused to an octahedral sheet of aluminum hydroxide. In the structure of MMT, octahedral aluminum ions are coordinated with oxygen and hydroxyl groups on one lamellar face, while tetrahedral silicon is covalently bonded with oxygen atoms on the other lamellar face. Adjacent layers are linked to one another by hydrogen bonding [23,24,25]. It has been demonstrated that fluoride ions can be easily incorporated into MMT due to its layered aluminosilicate structure and high surface area [26,27,28]. Thus, we herein employ fluorinated montmorillonite (FMMT) as a filler to the polymer matrix and as a source of fluoride ions for targeted release. The organofunctionalization of FMMT occurs with the intercalation of N-methylformamide (NMF) [29] to give the FMMT-NMF precursor, which undergoes the displacement of NMF with an acrylamide and yields the desired FMMT-acrylamide intercalation compound [30]. It is; therefore, desirable to develop FMMT-acrylamide compounds that can be readily grafted onto appropriate resin matrices to ultimately improve the mechanical properties of the resulting composite resin.

Thus, we herein report the development of a new light-curable sealant possessing suitable mechanical properties including the ability for fluoride release and recharge. We also demonstrate the good biocompatibility of this sealant, which should enable its application in a clinical environment.

## 2. Materials and Methods

FMMT was prepared by mixing MMT (10 g, 69866, Sigma-Aldrich, St. Louis, MO, USA) with H_2_O_2_ (40 mL, H1009, Sigma-Aldrich, St. Louis, MO, USA) in a 100 mL Teflon beaker and stirring at 50 °C for 30 min, followed by the addition of hydrogen fluoride (HF) (20 mL, diluted to 1 wt %; 339261, Sigma-Aldrich, St. Louis, MO, USA) and H_2_SO_4_ (10 mL 97%; 30743, Sigma-Aldrich, St. Louis, MO, USA) and stirring again at 50 °C for 30 min. The resulting FMMT was obtained by centrifugation. Residual HF was removed by the addition of a calcium d-gluconate solution (5 g in 100 mL deionized (DI) water, C8231, Sigma-Aldrich, St. Louis, MO, USA) and subjecting the resulting mixture to ultrasonication (3 × 30 min). FMMT was further washed with DI water (5 × 100 mL) to remove all calcium d-gluconate, and subsequently dried in an oven at 60 °C.

FMMT intercalated with *N*-methylformamide (FMMT–NMF) was prepared by the dispersion of a solution of FMMT in DI water (5 g in 5 mL) in *N*-methylformamide (60 mL, NMF; 473936, Sigma-Aldrich, St. Louis, MO, USA) followed by magnetic stirring at room temperature (25 °C) for 72 h [31]. The solid was collected following centrifugation (10 min at 4000 rpm) and then dried in a vacuum oven at 60 °C to obtain FMMT-NMF as a powder.

FMMT intercalated with acrylamide (FMMT-AAm) was synthesized by mixing a 12 wt % acrylamide solution (12 g acrylamide, A17157, Alfa Aesar, Ward Hill, MA, USA) in DI water (100 mL) with FMMT-NMF (2 g) under magnetic stirring at room temperature (25 °C) for 24 h. NaF (0.5 g) and a 5 M NaNO_3_ buffer (10 mL) were then added and the pH was adjusted to 4 by titration with 12 M HCl or 1 M NaOH [31]. The resulting acidic mixture was then magnetically stirred at room temperature (25 °C) for an additional 24 h followed by centrifugation at 4000 rpm for 10 min; the supernatant was discarded. The resulting product was dried in a vacuum oven at 60 °C to obtain the desired FMMT–AAm-NaF. Finally, the dried powder was ground and refined with a No. 635 sieve.

The fluoride-releasing composite resin (MLC-FMMT-containing light-curable composite resin) was finally prepared from FMMT-AAm-NaF and a light-curable polymer matrix. This polymer matrix was prepared by mixing bisphenol A-glycidyl methacrylate (bis-GMA), triethylene glycol dimethacrylate (TEGDMA), N,N-dimethyl-*p*-toluidine (DMPT), and camphorquinone in a weight ratio of 139:59:1:1 (all materials were purchased from Sigma-Aldrich, St. Louis, MO, USA). The previously-prepared FMMT-AAm-NaF powder acts as the inorganic filler in the MLC prepared using the light-curable polymer matrix to give 20 wt % FMMT–AAm-NaF in the composite resin. Subsequent polymerization was carried out using blue light with a wavelength of 460–510 nm (Litex 696, Dentamerica, City of Industry, CA, USA).

Characterization of the synthesized compounds was by X-ray diffractometry (XRD), Fourier-transform infrared (FTIR) spectroscopy, thermogravimetric analysis (TGA), and particle size distribution analysis. XRD was used to identify the crystal structure and to examine the change in planar spacing of the (001) plane in the montmorillonite lattice after intercalation with NMF and acrylamide (FMMT-NMF and FMMT-AAm-NaF). The prepared powders were mounted on a sample holder of an X-ray powder diffractometer (Miniflex, Rigaku, Tokyo, Japan) under Cu KαI radiation (*λ* = 0.15406 nm) with a Ni filter. The scan range was from 2° to 50° with a scan rate of 1°/min. Characteristic peaks were used to identify the crystal structure. The 2*θ* shift of the (001) plane of montmorillonite was used to evaluate the change in planar spacing.

Bonds between atoms can exhibit a variety of different vibrational modes that relay information regarding the functional groups present in organic molecules. Upon infrared excitation, different types of vibrations are produced at specific frequencies, whereby FTIR can be used to analyze the functional groups present when the dipole moment of the molecule is changed. In this study, the spectra of FMMT–NMF and FMMT–AAm-NaF were compared with those of pure NMF and AAm to confirm successful intercalation of the polymers into the layered structure.

TGA is a thermal analysis method in which the mass of a sample is measured over time as the temperature changes. In this study, the temperature of the sample was increased from 100 to 600 °C at a rate of 5 °C/min to compare the weight loss percentages of MMT, FMMT, FMMT–NMF, and FMMT–AAm-NaF at 600 °C to confirm the polymer content in the FMMT. Data were collected starting at 100 °C to remove the effect of moisture and for weight calibration following the complete removal of water/moisture.

To further characterize the filler component of the MLC, the size of the filler was investigated, as it is known to affect both the mechanical properties of the complex resin and the surface smoothness [32]. Therefore, a sample of FMMT–AAm-NaF (0.1 g) was added to DI water (2 mL) to measure the particle size using a light-scattering instrument (Zetasizer nano, Malvern Co., Malvern, Worcs, UK).

Subsequently, the mechanical properties (i.e., viscosity, curing depth, hardness, diametral tensile strength, flexural strength, and wear resistance) were examined. The viscosity (*η*) is defined as the shear stress (*τ*) divided by the shear strain rate (*γ*). The lower the viscosity of the unpolymerized composite resin, the more effectively it can enter the narrow gaps of pits and fissures [33]. The viscosity was assessed using a cone and plate rheometer (AR-1000 Rheometer, TA, New Castle, DE, USA), whereby the unpolymerized composite resin was placed on a horizontal plate and then a shallow cone supported by a torsion bar was laid on top. Data were collected at shear rates between 50 and 250 s^−1^.

The curing depth was also measured to determine the transmittance of the light-curable composite resin MLC. According to the ISO 6874:2015 guidelines, the curing depth should be at least 1.5 mm [34]. Therefore, a stainless steel mold with a diameter of 4 mm and depth of 6 mm was used. After filling the mold with the desired the material, blue light (*λ* = 460–510 nm; Litex 696, Dentamerica, City of Industry, CA, USA) was irradiated in one direction, and the curing depth was measured following demolding.

To examine the sample hardness, a stainless steel mold with a diameter of 6 mm and height of 3 mm was used. Following double-sided irradiation with blue light and subsequent demolding, the sample surfaces were wet-polished with silicon carbide paper from 400- to 1000-grit. The hardness test was performed using a diamond indenter with a 0.05 kg load and a 30 s dwell time. Measurements were performed in triplicate on the surface of each sample and the mean value was determined as the hardness. Ten mean values of ten samples in each group were calculated again to obtain the final mean value and the standard deviation of hardness. The Vickers hardness was tested using an HMV-2 tester (Shimadzu, Tokyo, Japan).

Compression and tensile strength are key properties of a dental composite resin since the resin must exhibit a sufficient resistance to brittleness in addition to being able to withstand repeated masticatory forces [35]. Thus, the diametral tensile strengths of 10 samples were evaluated following the New American Dental Association Specification No. 27 method [36]. Cylindrical samples were prepared in a stainless steel mold (3 mm high, 6 mm diameter) and polymerized as described above. The samples were chosen following visual confirmation that they did not contain any bubbles or defects on the surface; any flash present was removed using sandpaper (this process was also employed for the flexural strength and wear resistance tests described below). The test specimens were mounted vertically between disks of a universal testing machine (HDX, Instron, Norwood, MA, USA) with a 0.5 mm/min crosshead speed to calculate the diametral tensile strength using the following equation [37]:

$$\sigma_{t}=\frac{2P}{\pi DT}$$

where *σ_t_* is the tensile stress, *P* is the load at fracture, *π* is ratio of the circumference of a circle to its diameter, *D* is the diameter, and *T* is the thickness.

Composite resin commonly fails due to material fracture caused by external forces, and so the fracture resistance of a material should be evaluated using the flexural strength test [38]. Thus, to determine the flexural strength, we followed the guidelines specified in ISO 4049 [39]. A stainless steel mold (24 mm length × 2 mm height × 2 mm width) with a cross-sectional area of 4 mm^2^ was employed for this purpose. Following double-sided irradiation with blue light and subsequent demolding, the samples were placed on a three-point bending test device with a 20 mm distance between supports to ensure an equally distributed load. The universal testing machine was then operated at a 0.5 mm/min crosshead speed to determine the flexural strength (α) from the following equation:

$$\alpha =\frac{3FL}{2bh^{2}}$$

where *F* is the load (kN), *L* is the span length (cm), *h* is the sample thickness (cm), and *b* is the sample width (cm).

At present, no standard test method exists to evaluate the wear resistance of sealants. Thus, to determine the wear resistance, a self-designed acrylic mold (3 mm diameter, 20 mm height) was used and blue light was irradiated around the mold for 60 s to polymerize the MLC. A tribometer (UMT-2, CETR, Campbell, CA, USA) with a 10 N loading on number 400 silicon carbide grinding paper was used with a rotational speed of 100 rpm over 500 cycles. The wear resistance was then calculated by dividing the remaining weight by the original weight.

The fluoride release and recharge ability of the MLC was measured for the various samples using the commercially available Clinpro^TM^ (3M, Maplewood, MN, USA) as the control group. Six samples were selected for each set of materials. Samples were prepared using a mold (6 mm diameter, 2 mm height) and polymerization was carried out as described above. A continuous flow device was used to measure the release of fluoride. More specifically, each material was placed in DI water (5 mL), which was removed every 24 h and replaced with fresh DI water over 14 d. The fluoride concentration in the DI water was measured each day using ion chromatography (883, Metrohm, Herisau, AR, Switzerland) and calculated according to the following equation:

$$\frac{\mu gF}{cm^{2}}=ppm\;F\left(\frac{\mu gF}{mL}\right)*mL\left(volume\;of\;medium\;at\;unit\;time\right)*\frac{1}{surface\;area\;of\;specimen\;\left(cm^{2}\right)}$$

The fluoride ion release per day was then used to calculate the cumulative fluoride release. After 14 d of fluoride release, each sample was subjected to sonication (3 × 15 mL DI water for 10 min) to remove any residual fluoride ions on the surface of the MLC samples. The different groups of samples were then placed in 0.2% aqueous NaF (5 mL) at pH 7 for 1 min [31] to determine the recharge ability of the MLC samples containing varying amounts of FMMT-AAm-NaF. The samples were then removed, air-dried, and placed in DI water (5 mL) to repeat the fluoride ion release process over 7 d. The daily fluoride release from this 7 d process was also calculated to determine the cumulative fluoride release amount after recharging.

To determine the biocompatibility of our material, the composite resin was evaluated using a WST-1 assay with 3T3 cells and tested according to the ISO 10993–5 standard [40] with an extraction solution prepared according to the ISO 10993–12 standard [41]. The MLC (0.2 g/mL) was added to Dulbecco’s modified Eagle medium (DMEM) and incubated at 37 °C in a 5% CO_2_ atmosphere for 3 d. The 3T3 cells were then seeded in 96-well plates at a cell density of 1 × 10^4^ cells/well and incubated at 37 °C in a 5% CO_2_ atmosphere for 1 d. The DMEM was then replaced with the extraction solution and the samples were incubated for 3 d. For comparison, DMEM was used as the control group and 1% Triton X-100 was employed as the positive control group (without added MLC). After incubation, the plates were placed in an ELISA reader (Epoch 2, BioTek, Highland Park, VT, U.S.A) set to record the absorbance at 450 nm (with a reference filter at 600 nm) associated with Formazan formation. Since WST-1 binds to mitochondrial dehydrogenase to produce an orange formazan, a darker color will indicate the presence of greater numbers of metabolically active cells. As such, determination of the formazan concentration allows quantification of the number of cells [42].

Statistical analyses were performed from data collected and presented as the mean ± standard deviation (SD). The results were compared using the two-tailed Student’s t-test, whereby differences were considered as statistically significant when *p* < 0.05, represented by *. When *p* < 0.005, this is represented by ** and when *p* < 0.001, it is indicated with ***.

## 3. Results

### 3.1. Material Characterization (XRD, FTIR, TGA, and PS)

#### 3.1.1. XRD

**Figure 1:**
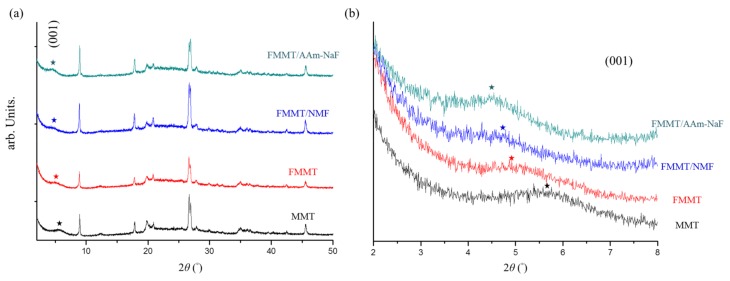
(**a**) XRD patterns of MMT, FMMT, FMMT-NMF, and FMMT-AAm-NaF from 2° to 50°; (**b**) low-angle XRD patterns (2°–8°) of MMT, FMMT, FMMT-NMF, and FMMT-AAm-NaF.

For montmorillonite and its derivatives, powder XRD was used to observe any changes in the crystal structure after modification. Initially, HF and H_2_SO_4_ were used to modify and convert MMT to FMMT. No significant changes in the XRD pattern were observed between these two samples (Figure 1a), however in the (001) low-angle plane, the position of 2*θ* changed from 5.58° to 4.85° (Figure 1b). Following the reaction of FMMT with NMF to give FMMT-NMF, 2*θ* was further shifted to 4.63°. Finally, FMMT-NMF was converted to the acrylamide, FMMT-AAm-NaF, and 2*θ* was again shifted, this time to 4.46°. The (001) plane angle is marked with a star.

#### 3.1.2. FTIR

**Figure 2:**
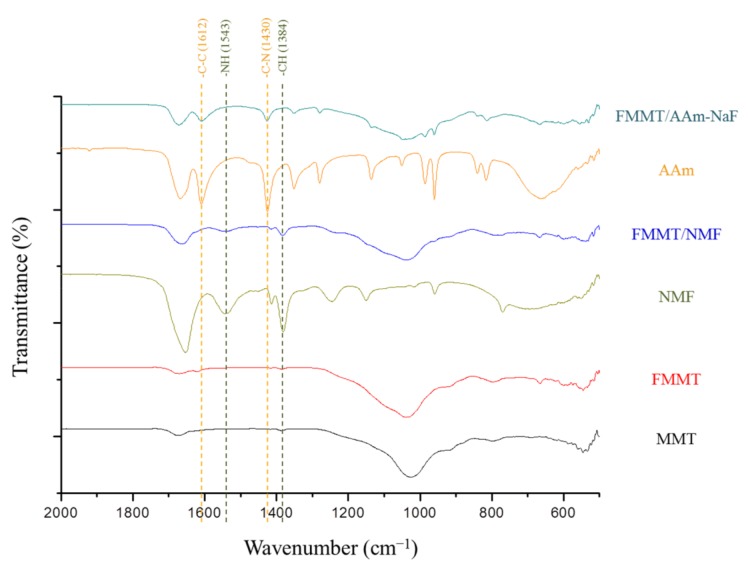
FTIR spectra (500–2000 cm^−1^) of MMT, FMMT, NMF, FMMT-NMF, AAm, and FMMT-AAm-NaF.

FTIR can be used to confirm that the modified FMMT-NMF and FMMT-AAm-NaF fillers do indeed contain these two polymers. Therefore, the filler materials were compared with pure NMF and AAm (Figure 2). As shown, the FTIR spectrum of NMF displays bands at 1384 cm^−1^ (–CH) and 1543 cm^−1^ (amide II) [43], which are characteristic of NMF and which are not observed in the spectra of either MMT or FMMT, but are present in that of FMMT-NMF. In addition, bands arising from the AAm polymer at 1621 cm^−1^ (–CC) and 1430 cm^−1^ (–CN) [44] were also observed for FMMT-AAm-NaF, but not for MMT or FMMT.

#### 3.1.3. TGA

**Figure 3:**
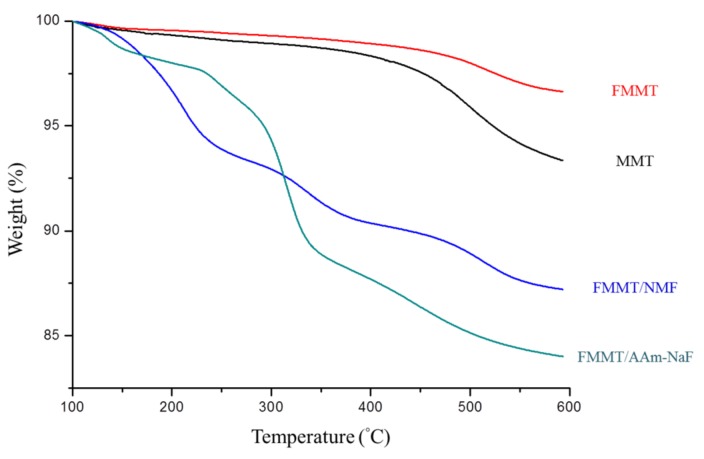
TGA patterns of MMT, FMMT, FMMT-NMF, and FMMT-AAm-NaF between 100 and 600 °C.

TGA was employed to confirm the polymer content in the filler (Figure 3). Due to the shielding effect of FMMT after the intercalation of NMF and replacement with AAm, the two polymers were not immediately decomposed by heating to the boiling points of these two polymers. Rather, decomposition was divided into multiple stages that were related to slow dissipation via different functional groups. The thermogravimetric curve of FMMT-NMF indicates that ~9.7% of NMF was present in FMMT following NMF insertion into the FMMT (001) plane. After replacement, the filler contained ~13.07% AAm.

#### 3.1.4. Particle Size

**Figure 4:**
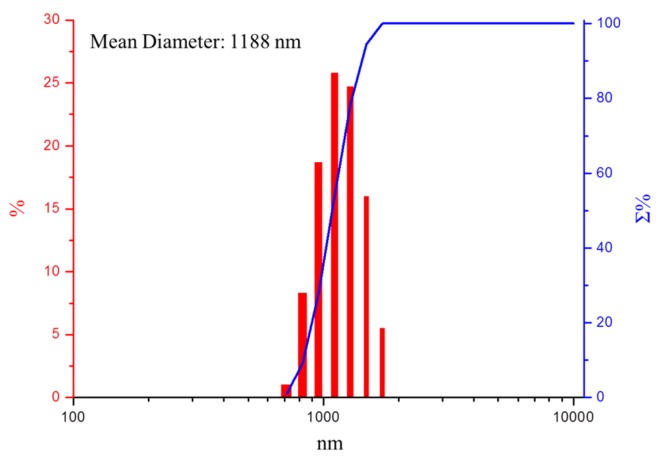
Particle size distribution (red column) and accumulation (blue line) pattern of the FMMT-AAm-NaF filler.

The size of the particles affects the mechanical properties and surface smoothness of the composite. Upon synthetic modification from MMT to FMMT-AAm-NaF, polymer insertion and aggregation after drying increased the particle size. The particle size of the FMMT-AAm-NaF filler employed for mixing with the polymer matrix to prepare the desired MLC was determined to be ~1188 nm (Figure 4).

### 3.2. Mechanical Analysis (Viscosity, Curing Depth, Hardness, Diametral Tensile Strength, Flexural Strength, and Wear Resistance)

#### 3.2.1. Viscosity

**Figure 5:**
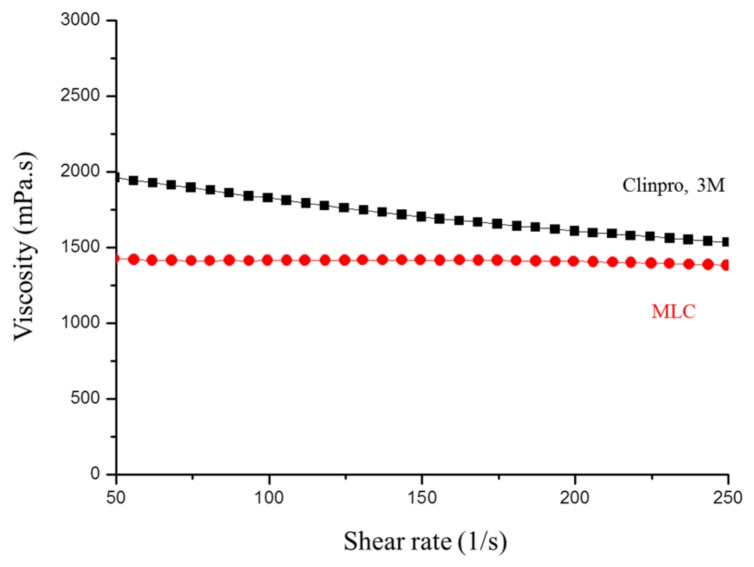
The viscosities of uncured MLC and Clinpro^TM^ with shear rates ranging from 50 to 250 s^−1^.

Determination of the viscosity of a composite resin allows us to obtain the flow properties of the material. Since pits and fissures are essentially microcracks or deep grooves in the tooth, the composite resin should exhibit good flow properties to allow its deep penetration into the inner layer of the tooth and effectively fill the tooth defects. For comparison prior to polymerization, Clinpro^TM^ was used as the control group. As shown in Figure 5, the composite resin containing 20 wt % FMMT–AAm-NaF (MLC) exhibited a slightly lower flowability than the commercial product.

#### 3.2.2. Curing Depth, Hardness, Diametral Tensile Strength, Flexural Strength, and Wear Resistance

The light penetration ability of the composite resin can be determined by measuring the curing depth of the composite resin; the stronger the light penetrating ability, the deeper the curing depth. As shown in Table 1, the light penetration ability of MLC is lower than that of Clinpro^TM^, although the average value is still greater than the ISO standard of 1.5 mm.

In addition, the hardness, diametral tensile strength, and flexural strength provide insight into the resistance of the composite resin to external stresses, such as punctures or pressure due to biting. As outlined in Table 1, the average values of the developed MLC in terms of these mechanical properties were lower than those of the 3M product with statistically significant differences.

Furthermore, in the wear resistance test, the weight remaining after the test was divided by the original weight and multiplied by 100 to obtain the percentage. It was found that the wear resistances of the MLC and Clinpro^TM^ were comparable, with the weight after the test being ~85% of the original weight. No statistically significant difference was observed in this case.

### 3.3. Oral Environment Simulation

#### 3.3.1. Fluoride Release Measurements

**Figure 6:**
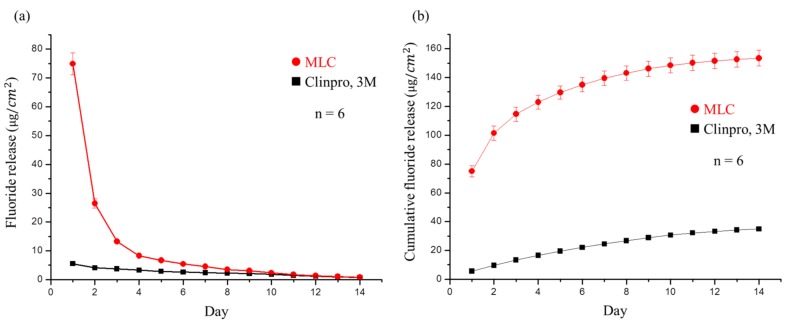
(**a**) Fluoride release per square centimeter of Clinpro^TM^ and MLC each day for 14 days. No significant difference was observed on day 14. (**b**) Cumulative fluoride release of Clinpro^TM^ and MLC each day for 14 days. Significant differences existed between both materials at all times during the trial.

As the release of fluoride ions is the focus of this study, we employed Clinpro^TM^ as the control group due to the claim by 3M that this product exhibits an effective fluoride ion release and recharge ability. Thus, Figure 6a shows a representation of fluoride release from the two samples (MLC and Clinpro^TM^) every 24 h over two weeks. As indicated, the developed MLC exhibited the largest amount of fluoride ion release in the initial three days, although the release per unit area dropped rapidly thereafter. The single-day release on the eighth day was similar to that of Clinpro^TM^, and was equal on the fourteenth day. We; therefore, confirmed that Clinpro^TM^ exhibits a suitable fluoride release ability as claimed, with a relatively slow decrease in release with time. However, its initial fluoride release rate was lower than that of MLC, as was its cumulative fluoride release (Figure 6b).

#### 3.3.2. Fluoride Recharge Measurements

**Figure 7:**
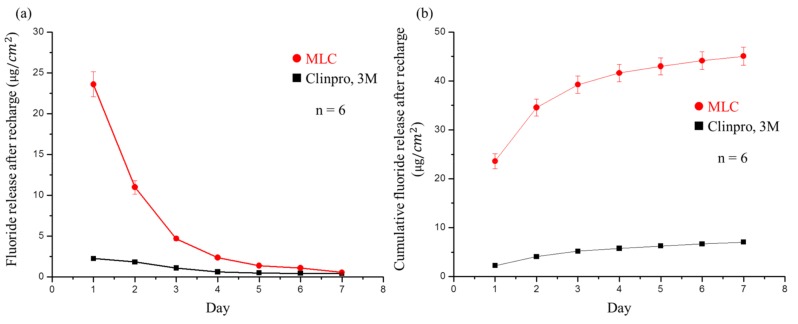
(**a**) Fluoride release after recharge per square centimeter of Clinpro^TM^ and MLC each day for seven days. Significant differences were observed between both materials at all times except on the seventh day. (**b**) Cumulative fluoride release from Clinpro^TM^ and MLC after recharge every day for seven days. Significant differences existed between both materials at all times.

The recharge of fluoride ions is also an important parameter to consider in the development of sealant materials, with the ultimate goal being for patients to use a fluoride mouthwash or fluoride toothpaste to supplement fluoride in the sealant. As shown in Figure 7a, the developed MLC exhibited 10 μg fluoride ion release in the first two days after recharge with a 0.2% NaF solution, far exceeding the 2.24 μg of Clinpro^TM^. As with the fluoride ion release measurements described above, the release concentration after recharge displays a steep downward slope, decreasing more rapidly than in the case of Clinpro^TM^, and on the fourth day, the slope becomes shallow with slower release. However, on the final day of the test (day seven), no significant difference was observed for Clinpro^TM^. As shown in Figure 7b, which indicates the cumulative amount of fluoride ion released after recharge, MLC exhibited a significantly higher cumulative fluoride ion release than the commercial product.

### 3.4. Biocompatibility

**Figure 8:**
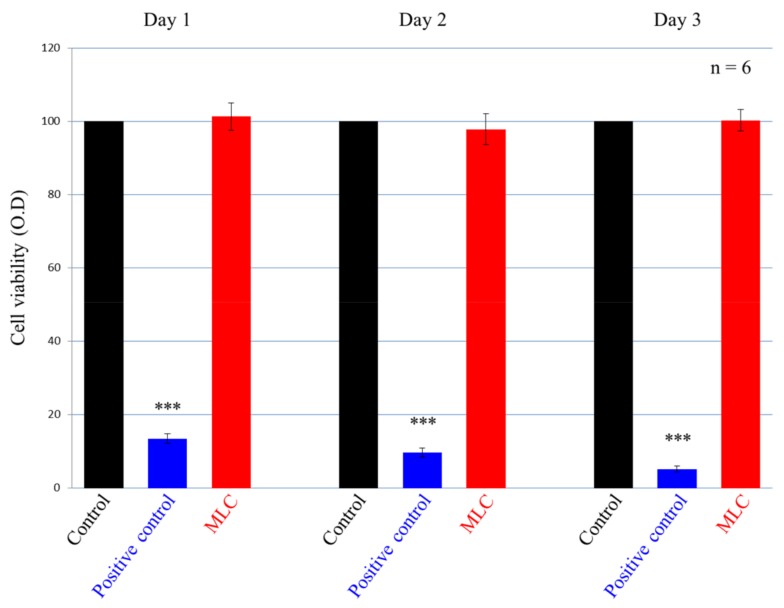
WST-1 assay of the control group, positive control group, and MLC extraction treated 3T3 cells at 24, 48, and 72 h. Only the positive control group was statistically significantly different from the other two groups (*** represents *p* < 0.001); the MLC was not significantly different from the control group.

The biocompatibility of the MLC was determined using 3T3 cells with the WST-1 assay. Thus, the cell viability was measured from days one to three, with the results expressed as a percentage of the cell viability (Figure 8). Pure medium was used as the control group and the positive control group was 1% Triton X-100. The cell viability of the experimental group did not show any significant difference compared to the control group. These results confirm that the MLC extraction solution did not produce cytotoxic effects or inhibit cell proliferation in 3T3 fibroblasts.

## 4. Discussion

The purpose of this study was to develop a new light-curable sealant possessing suitable mechanical properties and a good fluoride release and recharge ability. In the XRD analysis, it could be seen that after a series of modifications to MMT, the original crystal structure was retained, although the (001) plane spacing was enlarged upon the introduction of NMF into FMMT, and then again upon the replacement of NMF with acrylamide. The obtained 2*θ* angle was converted using Bragg’s law to obtain the change in (001) plane spacing of MMT. Thus, the original MMT of 5.58° corresponded to a spacing of 1.58 nm, while after the reaction with HF and H_2_SO_4_ to give FMMT, a spacing of 1.82 nm was calculated. Upon the introduction of NMF, the plane distance of FMMT–NMF increased to 1.91 nm (converted from the diffraction angle of 4.63°). In the final step where NMF was replaced with acrylamide, the (001) plane of FMMT-AAm-NaF expanded to 1.98 nm. Such expansion of the plane spacing is desirable since the modified MMT contains a larger surface area for bonding the fluoride ion, and also because the interlayer polymer adsorbs greater quantities of NaF to allow effective controlled release [45,46].

FTIR spectroscopy confirmed that the modified FMMT-NMF and FMMT-AAm-NaF fillers contained these two polymers, but it was not possible to confirm whether the polymer was coated on the outer layer of the FMMT or intercalated into the interlayer spacing. It was; therefore, necessary to further probe the system by TGA. In general, if the polymer is only coated on the outer layer of the inorganic material, it will be removed immediately when heating to the boiling point of the polymer. However, the weight shown in the TGA plot exhibited a stepwise decrease with increasing temperature. This difference is due to the shielding effect of the smectic clay, which prevents the polymer between the layers from being removed in a single step [47,48]. The combined results from XRD, FTIR, and TGA thereby confirm that MMT was successfully modified to give FMMT-AAm-NaF.

Previous studies have shown that the size of the filler in the composite resin is related to the mechanical properties and surface smoothness [32]. In our case, the average diameter obtained after machine analysis was ~1.2 μm, and we believe that this size of filler can be uniformly distributed in the composite resin to provide an appropriate mechanical strength along with a smooth surface. Such a particle size would not be expected to cause the filler to precipitate in the uncured resin.

When employed in a clinical setting, the sealants primarily fill the small cracks of pits and fissures present in teeth. As there is support from the surrounding teeth, the mechanical properties of the sealants do not need to be as high as those of other restoration materials. However, the viscosity of the MLC should be at least as good as or better than that of Clinpro^TM^, due to this commercial product being currently considered by the majority of dentists in the National Taiwan University Children’s Hospital to be the optimal material. In terms of the hardening depth, our light penetration was found to be significantly smaller than that of Clinpro^TM^, but it should be noted that this does not affect its clinical operation. According to previous studies, the majority of pits have a depth of no more than 1.05 mm, and the sealant prepared using this composite resin is in compliance with the ISO 6874:2015 specifications as long as it is >1.5 mm [34].

In terms of the micro hardness, diametral tensile strength, and flexural strength, MLC performs worse than the commercial product, but we speculate that because of the large amount of tiny bubbles produced in the composite resin when the filler and polymer matrix were mixed, even the samples selected for mechanical testing that contained no visible bubbles and burrs likely contained imperfections that were not visually observed. We did attempt degassing by ultrasonication and using a Heidelberg hemisphere for sample evacuation, but it was not possible to completely remove the tiny bubbles from the resin. These bubbles cause stress concentrations, which greatly reduce the mechanical properties of the MLC [49], and in particular for the diametral tensile strength and the flexural strength. If there is an opportunity to develop MLC as a commercial product, the bubbles inside the composite resin could be removed professionally, thereby yielding superior mechanical properties compared to those that can be obtained in the laboratory. In terms of the hardness, the filler present in Clinpro^TM^ is 6 wt % SiO_2_. Thus, although the selected weight ratio of the FMMT–AAm-NaF is 20 wt %, the hardness of MMT is significantly lower than that of SiO_2_ and; therefore, in addition to the influence of bubbles, the hardness of the inorganic filler must also be considered.

Subsequently, to simulate the oral environment, we tested the ability of MLC to release and recharge fluoride ions. More specifically, we found that fluoride ion release was high in the first three days. We believe that, in addition to the fluoride ions released from grafting to FMMT, additional fluoride is released by the NaF present in AAm. In this way, it can reasonably be explained that after the recharge process, the fluoride ion release concentration cannot reach such a high level. The single source of Clinpro^TM^ release of fluoride ions is tetrabutylammonium tetrafluoroborate (TBATFB) [50], whereas acrylamide was used in this study, which exhibits a poor fluoride recharge effect in terms of the polymer properties. We; therefore, believe that the source of MLC fluoride ion release after recharge is based on the incorporation of fluoride ions from NaF on FMMT. Although it is clearly shown in Figure 7b that the amount of fluoride release rapidly declines, this is not of concern, since patients generally use a fluoride-containing mouthwash or fluoride toothpaste twice a day, thereby giving two recharge opportunities each day. The fluoride ion release concentration of MLC in the oral cavity can; therefore, be maintained.

In the MLC extraction of the WST-1 assay, MLC was broken into small pieces according to ISO 10993–12 (making its surface area >60 cm^2^) and incubated at 37 °C for 24 h as an extract. A time period of 24 h was chosen since MLC exhibits its highest fluoride ion release on the first day. If the growth of 3T3 cells is not affected during the first day, then additional days of extraction should not be an issue. In addition, from Figure 8 it can be seen that our materials do not produce cytotoxicity and can; therefore, be used safely as sealant materials.

## 5. Conclusions

We herein reported the development of a fluoride–montmorillonite (FMMT) nanocomposite resin that exhibited the potential to provide sustained release of fluoride due to the strong adsorption of fluoride by montmorillonite. In addition, it was confirmed that MMT was successfully converted into FMMT-AAm-NaF (AAm = acrylamide) after a series of modifications, and that this material exhibited a dual fluoride ion release mechanism. The filler was mixed at a 20 wt % ratio into a polymer matrix containing bisphenol A-glycidyl methacrylate and triethylene glycol dimethacrylate as the composite resin of the sealant, and the properties of the prepared MLC (FMMT-containing light-curable composite resin) were tested. Although the majority of data recorded indicated that this material exhibited poorer mechanical properties than a commonly-employed commercial product, we believe that if the bubbles can be eliminated from our prepared resin, the mechanical properties will be significantly improved. Furthermore, simulation of the oral environment indicated that the prepared MLC exhibits excellent fluoride ion release and recharge properties, thereby rendering this a promising composite material that can be effectively used in the oral cavity to prevent dental caries. Moreover, a WST-1 assay also indicated that no cytotoxicity was presented by the MLC. We; therefore, believe that the MLC described herein has the potential to open an innovative window for effective sealants for application in the clinical treatment of dental pits and fissures.

## Figures and Tables

**Table 1:** Mechanical properties of developed MLC and the commercial product Clinpro^TM^ (n = 10).

Materials	Curing Depth (mm)	Micro Hardness (kgf/mm^2^)	Diametral Tensile Strength (MPa)	Flexural Strength (MPa)	Wear Resistance (%)
Clinpro^TM^	5.24 ± 0.05	17.9 ± 0.3	34.0 ± 1.3	86.1 ± 1.7	85.8 ± 1.6
MLC	4.18 ± 0.10 ***	15.9 ± 2.3 **	25.9 ± 1.9 ***	61 ± 3 ***	84 ± 3

** Represents *p* < 0.005, *** represents *p* < 0.001, unlabeled represents *p* > 0.05 (no significant difference).
